# Use of machine learning to identify risk factors for insomnia

**DOI:** 10.1371/journal.pone.0282622

**Published:** 2023-04-12

**Authors:** Alexander A. Huang, Samuel Y. Huang

**Affiliations:** 1 Northwestern University Feinberg School of Medicine, Chicago, IL, United States of America; 2 Virginia Commonwealth University School of Medicine, Richmond, VA, United States of America; Florida State University, UNITED STATES

## Abstract

**Importance:**

Sleep is critical to a person’s physical and mental health, but there are few studies systematically assessing risk factors for sleep disorders.

**Objective:**

The objective of this study was to identify risk factors for a sleep disorder through machine-learning and assess this methodology.

**Design, setting, and participants:**

A retrospective, cross-sectional cohort study using the publicly available National Health and Nutrition Examination Survey (NHANES) was conducted in patients who completed the demographic, dietary, exercise, and mental health questionnaire and had laboratory and physical exam data.

**Methods:**

A physician diagnosis of insomnia was the outcome of this study. Univariate logistic models, with insomnia as the outcome, were used to identify covariates that were associated with insomnia. Covariates that had a p<0.0001 on univariate analysis were included within the final machine-learning model. The machine learning model XGBoost was used due to its prevalence within the literature as well as its increased predictive accuracy in healthcare prediction. Model covariates were ranked according to the cover statistic to identify risk factors for insomnia. Shapely Additive Explanations (SHAP) were utilized to visualize the relationship between these potential risk factors and insomnia.

**Results:**

Of the 7,929 patients that met the inclusion criteria in this study, 4,055 (51% were female, 3,874 (49%) were male. The mean age was 49.2 (SD = 18.4), with 2,885 (36%) White patients, 2,144 (27%) Black patients, 1,639 (21%) Hispanic patients, and 1,261 (16%) patients of another race. The machine learning model had 64 out of a total of 684 features that were found to be significant on univariate analysis (P<0.0001 used). These were fitted into the XGBoost model and an AUROC = 0.87, Sensitivity = 0.77, Specificity = 0.77 were observed. The top four highest ranked features by cover, a measure of the percentage contribution of the covariate to the overall model prediction, were the Patient Health Questionnaire depression survey (PHQ-9) (Cover = 31.1%), age (Cover = 7.54%), physician recommendation of exercise (Cover = 3.86%), weight (Cover = 2.99%), and waist circumference (Cover = 2.70%).

**Conclusion:**

Machine learning models can effectively predict risk for a sleep disorder using demographic, laboratory, physical exam, and lifestyle covariates and identify key risk factors.

## Introduction

Sleep is critical to a person’s physical and mental health [1–6]. However, the prevalence of diagnosed sleep disorders among American patients has significantly increased over the past decade [1, 5, 7–10]. Sleep disorders are a broad categorization of disorders that encompass conditions that lead to difficulty falling asleep, poor sleep quality, early waking, circadian rhythm disorders, parasomnias, sleep-related movement disorders, and sleep-related breathing disorders [11–13]. This is particularly important as sleep disorders are a significant risk factor for diabetes, heart disease, obesity, and depression, leading to decreased quality of life and increased healthcare usage [14, 15]. Additionally, poor quality of sleep has been associated with decreased productivity at work and at school, increased stress, and decreased quality of life [16–19]. To combat the debilitating consequences of sleep disorders, a plethora of pharmacologic treatments have been introduced to the market and prescribed by physicians [20–26]. While medications have shown efficacy in decreasing sleep latency, significant side effects have been associated with these medications [27–32]. These include addiction, respiratory depression, decreased quality of sleep, and significant withdrawal symptoms when these medications are discontinued [21, 33–35]. Furthermore, due to the increasing prevalence of obstructive sleep apnea, continuous positive airway pressure (CPAP) machines are more regularly prescribed [27].

Despite recognition of sleep disorders as a strong contributor to increasing mortality and morbidity, little is known regarding specific risk factors that are strongly linked with increased probability of having sleep disorders. Given these limitations in the literature, we will leverage transparent machine-learning methods (Shapely Additive Explanations (SHAP) model explanations and model gain statistics) to identify pertinent risk-factors for sleep disorders and compute their relative contribution to model prediction of risk for sleep disorder; the NHANES 2017–2020 cohort, a large, nationally representative sample of US adults, will be used within this study.

### Methods

A retrospective, cross-sectional cohort study using the publicly available National Health and Nutrition Examination Survey (NHANES) was conducted in patients who completed the demographic, dietary, exercise, and mental health questionnaire and had laboratory and physical exam data. The acquisition and analysis of the data within this study was approved by the National Center for Health Statistics Ethics Review Board. Within this retrospective cohort, all data (medical records, survey information, demographic information) was fully anonymized before data analysis was carried out and all patients consented to their data being publicly available.

### Dataset and cohort selection

The National Health and Nutrition Examination Survey (NHANES 2017–2020) is a program designed by the National Center for Health Statistics (NCHS), which has been leveraged to assess the health and nutritional status of the United States population. The NHANES dataset is a series of cross-sectional, complex, multi-stage surveys conducted by the Centers for Disease Control and Prevention (CDC) on a nationally representative cohort of the United States population to provide health, nutritional, and physical activity data. In the present study, we analyzed adult (≥18 years old) patients in the NHANES dataset who completed the demographic, dietary, exercise, and mental health questionnaire and had laboratory and physical exam data.

### Assessment of sleep disorder

The medical conditions file was used to identify patients with a sleep disorder. Participants were asked: “Have you ever told a doctor or other healthcare professional that you have trouble sleeping?” Participants who answered “Yes” to this question were considered to have a sleep disorder within this study.

### Independent variable

Potential model covariates were identified within the demographics, dietary, physical examination, laboratory, and medical questionnaire datasets in NHANES. A total of 783 covariates were identified from the NHANES dataset. All covariates were extracted and merged with the sleep disorder indicator.

### Model construction and statistical analysis

Univariate logistic models, with a sleep disorder as the outcome, were used to identify covariates that were associated with a sleep disorder. Covariates that had a p<0.0001 on univariate analysis were included within the final machine-learning model. Utilizing univariable logistic models to do an initial filter of the 700+ covariates that were within the dataset was used to ensure that all covariates used within the machine learning models were strong independent covariates. Furthermore, this initial filtering allowed for physician review of risk factors that were clinically relevant. After initial filtering, model importance statistics from machine-learning models were used to identify pertinent risk factors.

Four machine-learning methods were carried out: XGBoost, Random Forest (RF), Adaptive Boost (ADABoost), and Artificial Neural Network (ANN). All machine-learning models were constructed using 10-fold cross validation. Cross validation was applied to only the training set. A train:test (80:20) was used to compute the final set of model fit parameters. The model fit parameters used in this study were accuracy, F1, sensitivity, specificity, positive predictive value, negative predictive value, and AUROC (Area under the receiver operator characteristic curve).

A grid search of hyperparameters for the XGBoost, Random Forest, and Adaptive Boost methods was conducted. Trees were searched between 200 and 2000 at 100 tree increments, with the optimal number being 600 trees for all models. The artificial neural network was comprised of an input layer with hidden layers and a scalar output layer. Additionally, the ReLu function at each hidden layer and a Sigmoid function at the output layer was used. The hyperparameters were determined by optimal accuracy across a grid search of 2–10 hidden layers, 128–1024 for hidden layer dimensions, and 64–512 for batch size. The hyperparameters that were most optimal were 4 hidden layers, 256 hidden layer dimensions, and 64 for the batch size.

The machine learning model XGBoost was used due to its prevalence within the literature as well as its increased predictive accuracy in healthcare prediction. Furthermore, XGBoost was chosen as the most optimal model based upon the mean AUROC: ($AUROC_{XGBoost}=0.87>AUROC_{ADABoost}=0.84>AUROC_{ANN}=0.83>AUROC_{RF}=0.82$, p<0.01).

### Model feature importance statistics and SHAP visualization

Model covariates were ranked according to the Gain, Cover, and Frequency to identify risk factors for a sleep disorder. The Gain is the relative contribution of the feature within the model. The Cover is the number of observations related to this feature that were present. The Frequency is the percentage of times the feature occurs in the trees of the machine-learning model. The Gain statistic was chosen as the method to rank features based upon feature importance due to its ease of interpretation: the proportion the covariate contributed to the final prediction.

SHAP explanations were utilized to visualize the continuous covariates with the strongest relationship between the potential risk factors and a sleep disorder.

## Results

**Fig 1:**
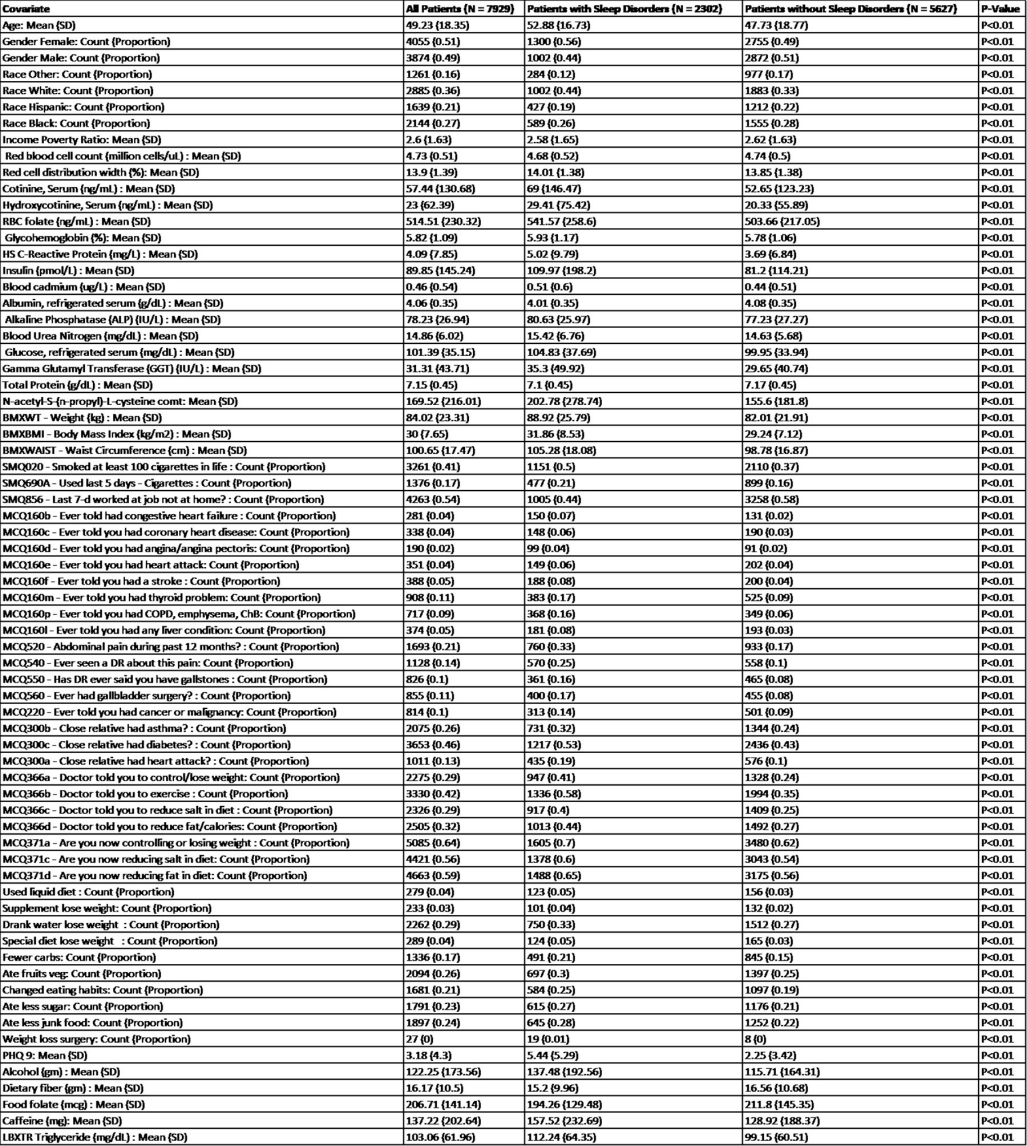
Demographic information and disease characteristics. Descriptive statistics for demographic characteristics and all covariates within the machine learning model, stratified by whether patients had a sleep disorder. Covariates with SMQ or MCQ labeled in front of it were asked the question written; responses were numeric (integer number) for SMQ and binary (yes, no) for MCQ. Abbreviations: DR = Doctor.

**Fig 1** shows of the 7,929 patients that met the inclusion criteria in this study, 4,055 (51% were female, 3,874 (49%) were male. The mean age was 49.2 (SD = 18.4), with 2,885 (36%) White patients, 2,144 (27%) Black patients, 1,639 (21%) Hispanic patients, and 1,261 (16%) patients of another race. A total of 2,302 (29%) of patients had a sleep disorder.

**Fig 2:**
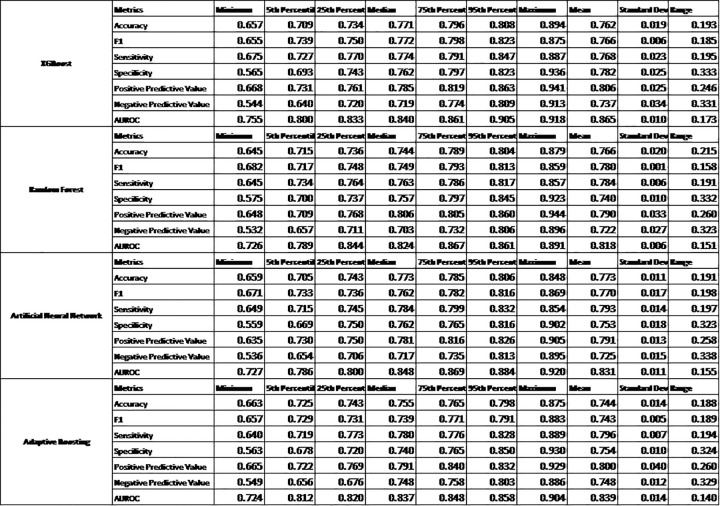
Comparison of different machine learning models. Comparison of four machine learning models (XGBoost, Random Forest, Artificial Neural Network, Adaptive Boosting) using the model statistics computed from the 20% test set: Accuracy, F1, Sensitivity, Specificity, Positive Predictive Value, Negative Predictive Value, and AUROC.

**Fig 3:**
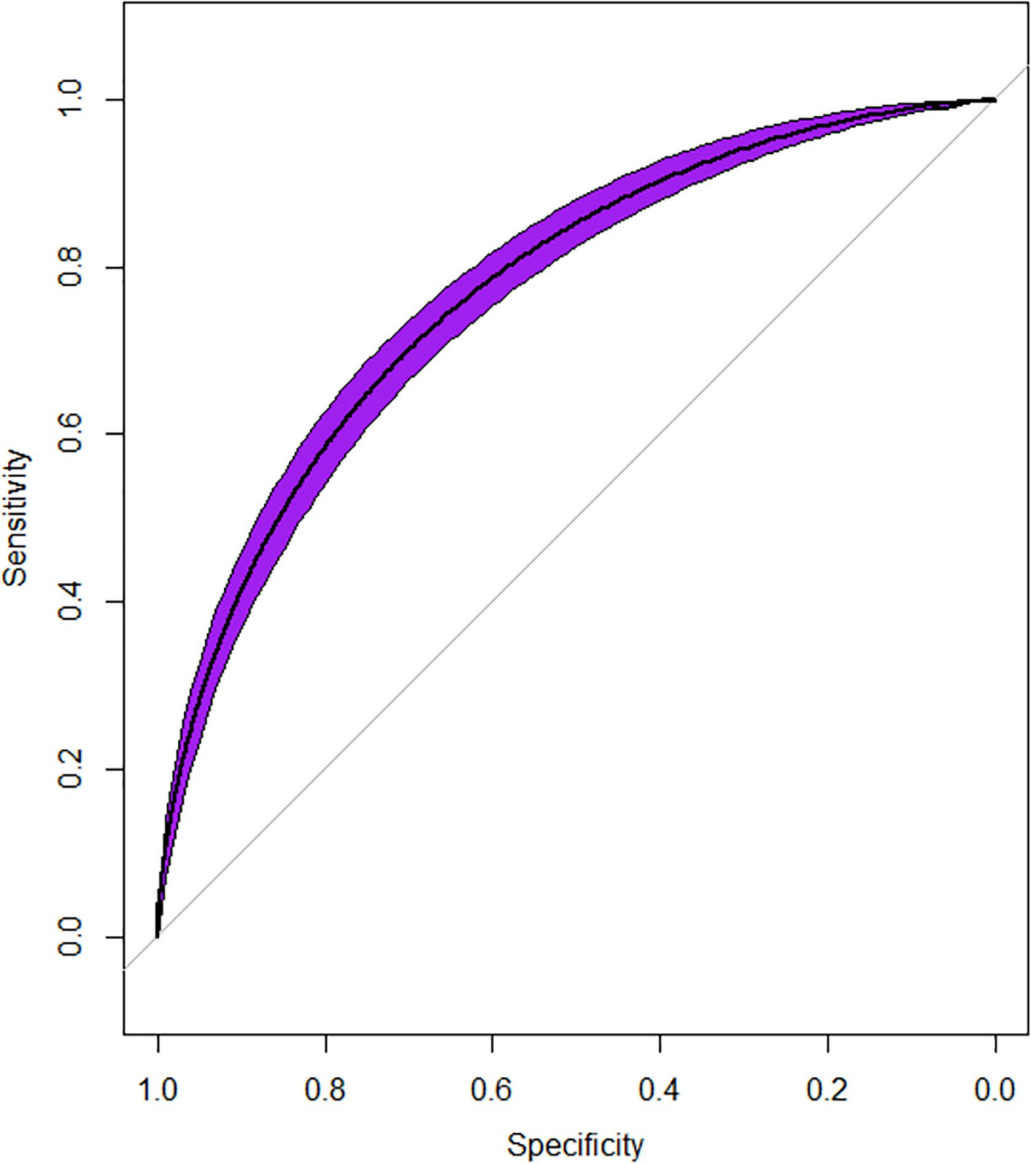
Receiver operator characteristic curve and model statistics. The Receiver operating characteristic curve for the machine-learning model predicting a sleep disorder. AUROC = 0.87.

**Fig 2** shows the comparison of different machine learning models that led to XGBoost being chosen for having the highest mean AUROC: ($AUROC_{XGBoost}=0.87>AUROC_{ADABoost}=0.84>AUROC_{ANN}=0.83>AUROC_{RF}=0.82$, p<0.01). The machine learning model had 64 out of a total of 684 features that were found to be significant on univariate analysis (P<0.0001 used). These were fitted into the XGBoost model and an AUROC = 0.87, Sensitivity = 0.77, Specificity = 0.78 were observed in **Fig 3**.

**Fig 4:**
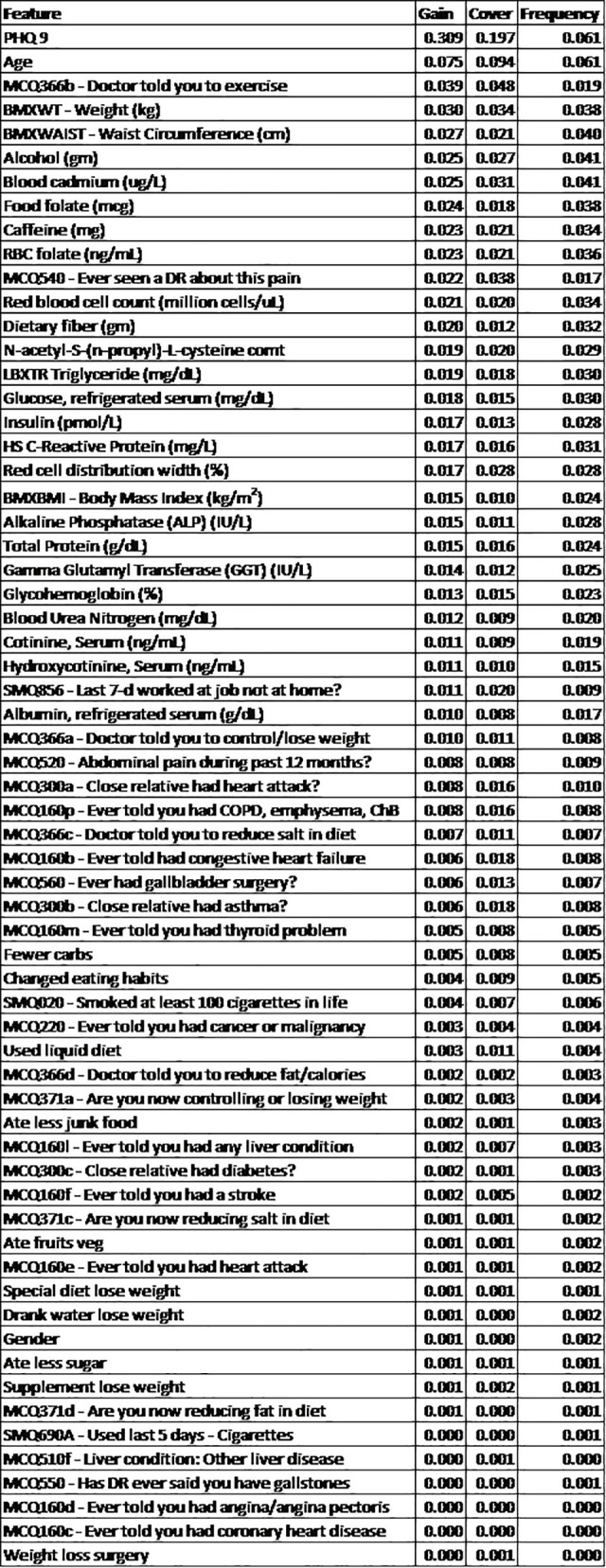
Model gain statistics. The Gain, Cover, and Frequency of all covariates within the XGBoost model. The Gain represents the relative contribution of the feature to the model and is the most important metric of model importance within this study. Covariates ordered according to the Gain statistic.

**Fig 5:**
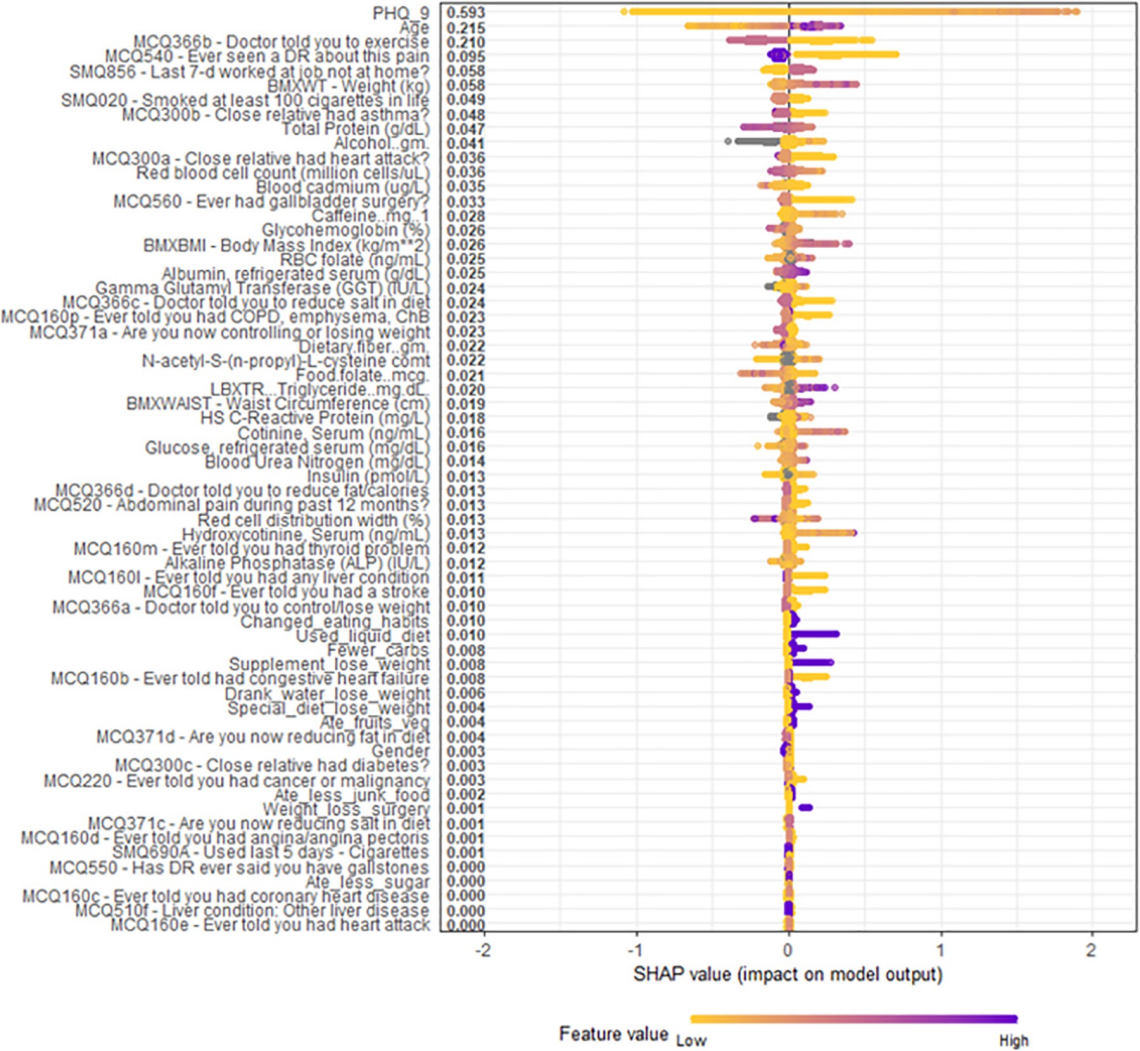
Overall SHAP explanations. SHAP explanations, purple color representing higher values of the covariate while yellow representing lower values of the covariate. X-axis is the change in log-odds for a sleep disorder. Covariates ordered according to the Gain statistic. Covariates with SMQ or MCQ labeled in front of it were asked the question written; responses were numeric (integer number) for SMQ and binary (yes, no) for MCQ. Abbreviations: DR = Doctor.

**Fig 4** shows the top four highest ranked features by cover, a measure of the percentage contribution of the covariate to the overall model prediction, were the Patient Health Questionnaire depression survey (PHQ-9) (Cover = 31.1%), age (Cover = 7.54%), physician recommendation of exercise (Cover = 3.86%), weight (Cover = 2.99%), and waist circumference (Cover = 2.70%). **Fig 5** shows the overall SHAP explanations for the covariates showing the PHQ-9, age, physician recommendation of exercise, weight, and waist circumference have the highest contribution to the model.

**Fig 6:**
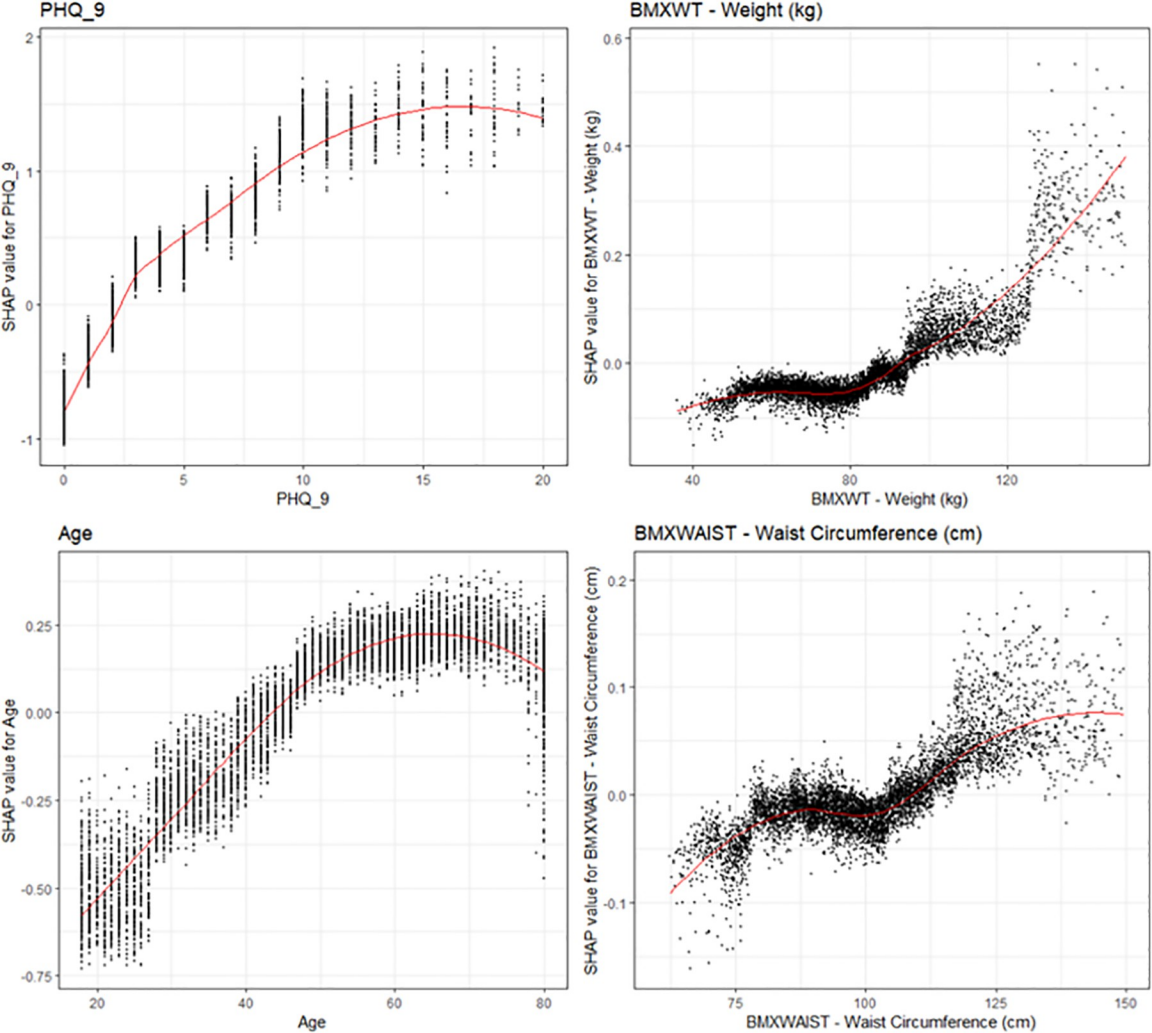
SHAP explanations for the Top 4 continuous covariates. SHAP explanations, covariate value on the x-axis, change in log-odds on the y-axis, red line represents the relationship between the covariate and log-odds for CAD, each black dot represents an observation. Covariates: top left–PHQ-9, top right–Body weight, bottom left–patient age, bottom right–waist circumference.

SHAP visualizations were conducted for the top four continuous covariates by model cover (**Fig 6**). We observed that increased PHQ-9 scores were strongly linked to the odds of a sleep disorder. Each increase in PHQ-9 score is associated with increased odds of a sleep disorder up to around a PHQ-9 score of 11, at which the odds of sleep disorder no longer increase with increased PHQ-9 score. Additionally, we observed a curvilinear relationship between weight and odds of a sleep disorder. There is no significant increase in odds of a sleep disorder with increasing weight for patients weighing under 80 kg, but after 80 kg, increased weight is associated with significantly increased odds of a sleep disorder. Furthermore, age was found to be a significant risk factor for a sleep disorder, with odds of a sleep disorder increasing between age 20 until age 60, at which point there does not appear to be an increase in sleep disorder with increasing age. Lastly, there is a strong relationship between waist circumference and a sleep disorder. There is no significant increase in odds of a sleep disorder with increasing waist circumference until after 100cm, at which there is a significant increase in odds of a sleep disorder with increasing waist circumference.

**Fig 7:**
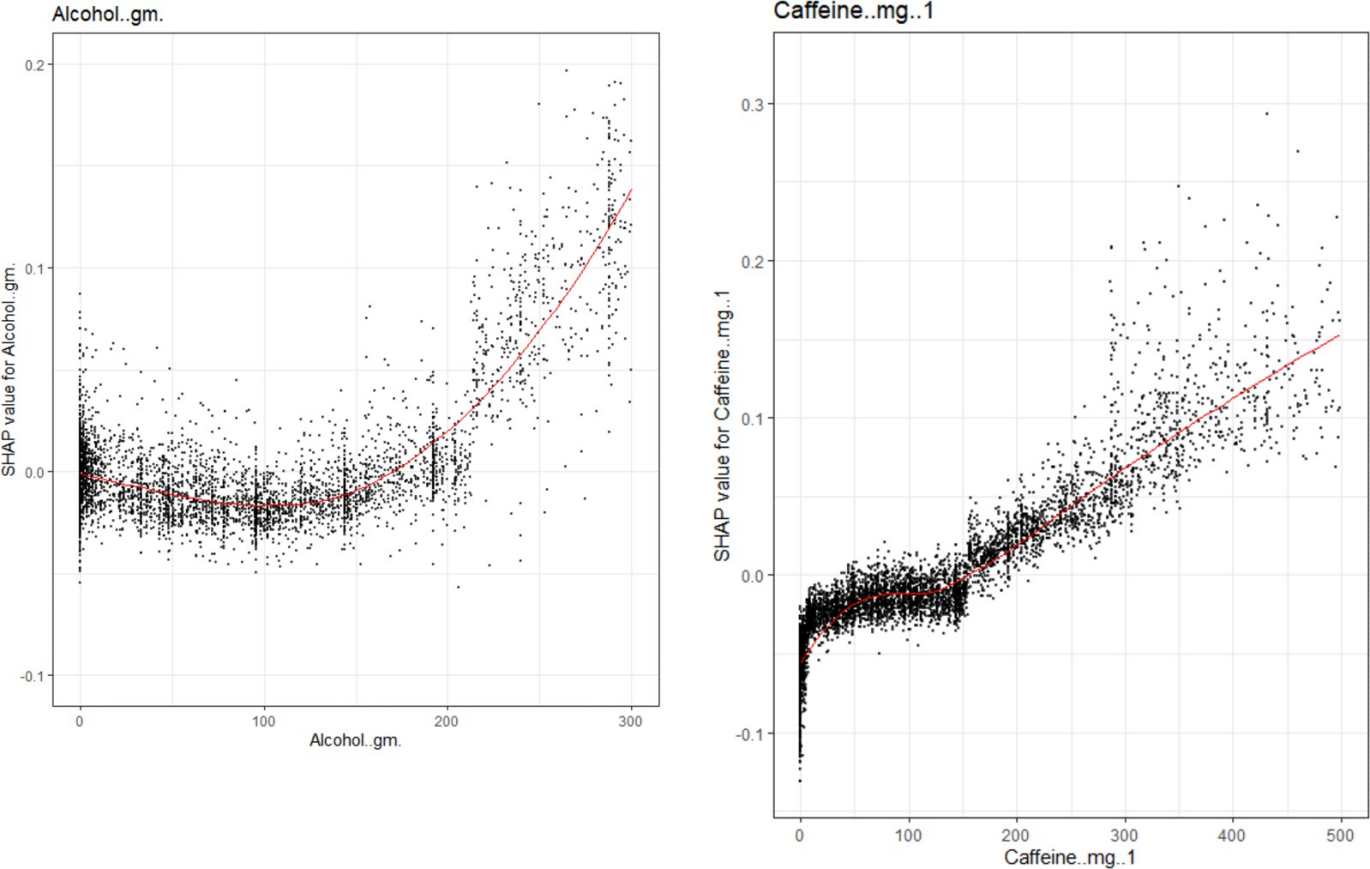
**a**: Covariates of interest to evaluate sensibility of the model. SHAP explanations for the relationship between Alcohol and odds of a sleep disorder. Covariate value on the x-axis, change in log-odds on the y-axis, red line represents the relationship between the covariate and log-odds for a sleep disorder, each black dot represents an observation. **b:** SHAP explanations for the relationship between Caffeine intake and odds of a sleep disorder. Covariate value on the x-axis, change in log-odds on the y-axis, red line represents the relationship between the covariate and log-odds for a sleep disorder, each black dot represents an observation.

**Fig 7A** show SHAP explanations for the XGBoost model and show a positive relationship between each gm of alcohol use and odds of a sleep disorder. Likewise **Fig 7B** show a positive relationship between each mg of caffeine and odds of a sleep disorder.

## Discussion

In this retrospective, cross sectional cohort of United States adults, a machine learning model utilizing demographic, laboratory, physical examination, and lifestyle questionnaire data had strong predictive accuracy (AUROC = 0.87). The greatest predictors for a sleep disorder included depression (PHQ-9), weight, age, and waist circumference.

Prior studies have accurately predicted the presence of sleep disorders using machine-learning methods from a variety of datasets using numerous machine-learning methods [36–38]. Short-term insomnia detection was conducted using a single-channel sleep Electrooculography. Furthermore, natural language processing on 18,901 tweets was conducted to find correlations between words related to insomnia and negative health information [38–40]. Furthermore, a comparative study of 15 machine learning algorithms identified 14 main factors for the prediction of insomnia, identifying that vision problems, mobility problems, and sleep disorders were significantly related to insomnia [38, 39]. These studies highlight the utility of machine learning models in identifying patients at risk for sleep disorders. What our study adds to the literature is a large dataset (N = 7,929) and a diverse wealth of potential covariates (700+ covariates) to study how lifestyle, diet, demographic, and medical covariates are able to predict insomnia.

The visualizations completed for the top four continuous covariates were concordant with current literature: there is strong epidemiological evidence that sleep problems are heavily linked with depression. Multiple papers have found difficulty falling asleep and decreased hours of sleep with increased depression [41–53]. Additionally, depression has bene linked to lower quality sleep and increased day time exhaustion [31, 34, 46, 54, 55]. There is also strong literature evidence for the link between weight and sleep disorders [4]. There is epidemiological evidence for the relationship between increased age and increased sleep disorders, older age has been associated with increased sleep latency, decreased time spent in rapid eye movement (REM) sleep and stage-3 sleep, and increased frequency of waking up during the night [56–62]. Furthermore, increased caffeine usage has been found to be linked with difficulty falling asleep, decreased time falling asleep, and decreased quality of sleep [63]. Additionally, increased alcohol is associated with sleep disorders, leading to decreased sleep latency and potential physiologic need for alcohol as a depressant to allow for sleep in multiple patients [64–66].

Since visualizations for risk factors match literature relationships, we have increased confidence that the machine learning model is able to capture the actual physiological relationships of these covariates. These transparent machine-learning tools allow for increased confidence that these algorithms are picking up true signal within these covariates to predict the presence of a sleep disorder rather than just replicating potential biases stemming from systemic data-quality errors that are present within the dataset. Additionally, these SHAP visualizations allow us to interpret that the increase predictive power of these machine-learning methods is associated with the ability for these non-parametric methods to more accurately capture the non-linear interactive relationship between the covariates, rather than just over-fitting the model to get increased accuracy.

The greatest strength of this algorithmic method for identification of the covariates is the ability to search through hundreds of covariates systematically without relying upon judgment form the researcher, which may be muddled by potential personal biases. This method also allows for the ranking of the relative importance of each of these covariates through the cover statistic, which allows us to obtain the relative contribution to the prediction each covariate has and thus infer from there an estimate for the relative contribution to true risk for a sleep disorder that each patient has. Another strength is that after these covariates are selected and the model built, SHAP visualizations can be used to make sure that each of the covariate either matches current literature understandings of the covariate’s association with a sleep disorder or in the case of a discrepancy, allow researchers to validate the plausibility of this feature and then evaluate for potential errors in data-quality.

A potential weakness to this machine-learning analysis is the necessity of the retrospective nature of this cohort. The covariates that were selected within this study will be better at predicting risk for a sleep disorder for this cohort than for other cohorts. However, this was limited by the use of training: testing sets to be able to minimize the errors that come with overfitting. Furthermore, visualizations of SHAP allow researchers to test for physiologic plausibility of each of these covariates and allows for effective analysis by researchers of whether these effects are due to true signal or if they are just noise that may be contributing to a type-1 error.

Given the analysis of the strengths and weaknesses of these methods, we argue that use of machine-learning methods can be an effective first step in the identification of risk-factors that can then be further selected by clinicians based upon the specific clinical presentation.

### Limitations

This study has several strengths and weaknesses. We utilized the NHANES dataset, which is a retrospective cohort, carrying the limitations of retrospective studies. However, this study allows for the selection of a large cohort, evaluation of data quality, and due to the publicly available nature of the cohort, allows for increased replication and follow-up studies based upon the same cohort. Furthermore, the cohort relied on surveys to obtain the outcome of interest (a sleep disorder requiring medical attention) as well as the dietary and lifestyle information. More accurate measurements may have been achieved with prospective studies with automated measurement of foods. However, self-reported survey information allows for the volume of participants to be included within this study. Another weakness was the voluntary nature of this cohort, with participants choosing to opt into the study instead of being randomly selected. This may artificially select a different cohort that may significantly differ from the population. However, our analysis found a demographically diverse population, so these results may still be generalizable to other cohorts.

### Conclusion

Machine learning models can effectively predict risk for a sleep disorder using demographic, laboratory, physical exam, and lifestyle covariates and identify key risk factors. Depression, age, weight, and waist circumference were the strongest predictors of sleep disorder.

## References

[1] Wang J, Ren X. Association Between Sleep Duration and Sleep Disorder Data from the National Health and Nutrition Examination Survey and Stroke Among Adults in the United States. Med Sci Monit. 2022;28:e936384. Epub 20220703. doi: 10.12659/MSM.936384 ; PubMed Central PMCID: PMC9261468. 35780293 PMC9261468

[2] Wolf C, Wolf S, Weiss M, Nino G. Children’s Environmental Health in the Digital Era: Understanding Early Screen Exposure as a Preventable Risk Factor for Obesity and Sleep Disorders. Children (Basel). 2018;5(2). Epub 20180223. doi: 10.3390/children5020031 ; PubMed Central PMCID: PMC5836000. 29473855 PMC5836000

[3] Guo Q, Xie W, Peng R, Ma Y, Chong F, Wang Y, et al. A Dose-Response Relationship Between Sleep Duration and Stroke According to Nonhealth Status in Central China: A Population-based Epidemiology Survey. J Stroke Cerebrovasc Dis. 2019;28(7):1841–52. Epub 20190507. doi: 10.1016/j.jstrokecerebrovasdis.2019.04.016 . 31076320

[4] Piepoli MF. Editor’s Presentation Benefit of healthy lifestyle on cardiovascular risk factor control: Focus on body weight, exercise and sleep quality. Eur J Prev Cardiol. 2019;26(12):1235–8. doi: 10.1177/2047487319861847 . 31298111

[5] Carriedo-Diez B, Tosoratto-Venturi JL, Canton-Manzano C, Wanden-Berghe C, Sanz-Valero J. The Effects of the Exogenous Melatonin on Shift Work Sleep Disorder in Health Personnel: A Systematic Review. Int J Environ Res Public Health. 2022;19(16). Epub 20220817. doi: 10.3390/ijerph191610199 ; PubMed Central PMCID: PMC9408537. 36011832 PMC9408537

[6] Sivertsen B, Hysing M, Harvey AG, Petrie KJ. The Epidemiology of Insomnia and Sleep Duration Across Mental and Physical Health: The SHoT Study. Front Psychol. 2021;12:662572. Epub 20210614. doi: 10.3389/fpsyg.2021.662572 ; PubMed Central PMCID: PMC8236531. 34194368 PMC8236531

[7] Shapiro CM, Dement WC. ABC of sleep disorders. Impact and epidemiology of sleep disorders. BMJ. 1993;306(6892):1604–7. doi: 10.1136/bmj.306.6892.1604 ; PubMed Central PMCID: PMC1678030. 8329929 PMC1678030

[8] Trosman I, Ivanenko A. Classification and Epidemiology of Sleep Disorders in Children and Adolescents. Child Adolesc Psychiatr Clin N Am. 2021;30(1):47–64. Epub 20201020. doi: 10.1016/j.chc.2020.08.002 . 33223068

[9] Bliwise DL. Epidemiology of Age-Dependence in Sleep Disordered Breathing (Sdb) in Old Age: The Bay Area Sleep Cohort (Basc). Sleep Med Clin. 2009;4(1):57–64. doi: 10.1016/j.jsmc.2008.11.004 ; PubMed Central PMCID: PMC2726445. 20161180 PMC2726445

[10] Kerkhof GA. Epidemiology of sleep and sleep disorders in The Netherlands. Sleep Med. 2017;30:229–39. Epub 20161105. doi: 10.1016/j.sleep.2016.09.015 . 28215254

[11] Ancoli-Israel S. Epidemiology of sleep disorders. Clin Geriatr Med. 1989;5(2):347–62. . 2665916

[12] Partinen M. Epidemiology of sleep disorders. Handb Clin Neurol. 2011;98:275–314. doi: 10.1016/B978-0-444-52006-7.00018-6 . 21056193

[13] Shamsadini A, Amizadeh M, Motamed S, Rajabi F. Epidemiology of Sleep Disorders Among Adolescents in Kerman, Iran, in 2019. Soc Work Public Health. 2022:1–6. Epub 20220613. doi: 10.1080/19371918.2022.2089310 . 35695466

[14] Pluzhnikov MS, Blotskii AA. [Epidemiology and laser correction of chronic diseases observed in snoring and obstructive sleep apnea]. Vestn Otorinolaringol. 2002;(3):12–5. . 12227021

[15] Rainer MA, Palmer PH, Xie B. Sleep Duration and Chronic Disease Among Older Native Hawaiians or Other Pacific Islanders and Asians: Analysis of the Behavioral Risk Factor Surveillance System. J Racial Ethn Health Disparities. 2022. Epub 20220915. doi: 10.1007/s40615-022-01409-0 . 36109435

[16] You Y, Zhong X, Liu G, Yang Z. Automatic sleep stage classification: A light and efficient deep neural network model based on time, frequency and fractional Fourier transform domain features. Artif Intell Med. 2022;127:102279. Epub 20220309. doi: 10.1016/j.artmed.2022.102279 . 35430040

[17] Jeong E, Cha KS, Shin HR, Kim EY, Jun JS, Kim TJ, et al. Corrigendum to "Alerting network alteration in isolated rapid eye movement sleep behavior disorder patients with mild cognitive impairment" [Sleep Med. 89 (2022) 10–18/SLEEP-D-21-00679]. Sleep Med. 2022;98:87–8. Epub 20220705. doi: 10.1016/j.sleep.2022.06.019 . 34864507

[18] Shah A, Turkistani A, Luenam K, Yaqub S, Ananias P, Jose AM, et al. Is Shift Work Sleep Disorder a Risk Factor for Metabolic Syndrome and Its Components? A Systematic Review of Cross-Sectional Studies. Metab Syndr Relat Disord. 2022;20(1):1–10. Epub 20211008. doi: 10.1089/met.2021.0070 . 34637354

[19] Vinstrup J, Jakobsen MD, Andersen LL. Poor Sleep Is a Risk Factor for Low-Back Pain among Healthcare Workers: Prospective Cohort Study. Int J Environ Res Public Health. 2020;17(3). Epub 20200205. doi: 10.3390/ijerph17030996 ; PubMed Central PMCID: PMC7036951. 32033339 PMC7036951

[20] Johnson EO, Roehrs T, Roth T, Breslau N. Epidemiology of alcohol and medication as aids to sleep in early adulthood. Sleep. 1998;21(2):178–86. doi: 10.1093/sleep/21.2.178 . 9542801

[21] Merino D, Gerard AO, Van Obberghen EK, Ben Othman N, Ettore E, Giordana B, et al. Medications as a Trigger of Sleep-Related Eating Disorder: A Disproportionality Analysis. J Clin Med. 2022;11(13). Epub 20220704. doi: 10.3390/jcm11133890 ; PubMed Central PMCID: PMC9267629. 35807172 PMC9267629

[22] Bergman E, Loyttyniemi E, Myllyntausta S, Rautava P, Korhonen PE. Temporal changes in self-reported sleep quality, sleep duration and sleep medication use in relation to temporal changes in quality of life and work ability over a 1-year period among Finnish municipal employees. J Sleep Res. 2022:e13605. Epub 20220415. doi: 10.1111/jsr.13605 . 35429092 PMC9787037

[23] Forthun I, Waage S, Pallesen S, Moen BE, Bjorvatn B. A shift to something better? A longitudinal study of work schedule and prescribed sleep medication use in nurses. Occup Environ Med. 2022. Epub 20220620. doi: 10.1136/oemed-2022-108251 PMC9606542 35725298

[24] Lambek R, Thomsen PH, Sonuga-Barke EJS, Jennum P, Sorensen AV. The Association between Sleep Problems and Neuropsychological Deficits in Medication-naive Children with ADHD. Behav Sleep Med. 2022;20(4):429–41. Epub 20210603. doi: 10.1080/15402002.2021.1931222 . 34081546

[25] Leite FMC, Xavier JCS, Silva RP, Wandekoken KD, Tavares FL, Amorim MHC. Prevalence and factors associated with the use of sleep-inducing medication among women receiving Primary Health Care: a cross-sectional study in Vitoria, Espirito Santo, Brazil, 2014. Epidemiol Serv Saude. 2022;31(1):e2021347. Epub 20220420. doi: 10.1590/S1679-49742022000100016 . 35475997

[26] Masse M, Henry H, Cuvelier E, Pincon C, Pavy M, Beeuwsaert A, et al. Sleep Medication in Older Adults: Identifying the Need for Support by a Community Pharmacist. Healthcare (Basel). 2022;10(1). Epub 20220113. doi: 10.3390/healthcare10010147 ; PubMed Central PMCID: PMC8775744. 35052310 PMC8775744

[27] Celik Y, Balcan B, Peker Y. CPAP Intervention as an Add-On Treatment to Lipid-Lowering Medication in Coronary Artery Disease Patients with Obstructive Sleep Apnea in the RICCADSA Trial. J Clin Med. 2022;11(1). Epub 20220105. doi: 10.3390/jcm11010273 ; PubMed Central PMCID: PMC8745784. 35012012 PMC8745784

[28] Darchia N, Campbell IG, Basishvili T, Eliozishvili M, Tchintcharauli T, Oniani N, et al. Longitudinal assessment of NREM sleep EEG in typically developing and medication-free ADHD adolescents: first year results. Sleep Med. 2021;80:171–5. Epub 20210201. doi: 10.1016/j.sleep.2021.01.052 . 33601229

[29] Forthun I, Waage S, Pallesen S, Moen BE, Bjorvatn B. Sleep medication and melatonin use among Norwegian nurses—A cross-sectional study. Nurs Open. 2022;9(1):233–44. Epub 20210917. doi: 10.1002/nop2.1057 ; PubMed Central PMCID: PMC8685790. 34534412 PMC8685790

[30] Full KM, Pusalavidyasagar S, Palta P, Sullivan K, Shin JI, Gottesman RF, et al. Associations of late-life sleep medication use with incident dementia in the Atherosclerosis Risk in Communities (ARIC) Study. J Gerontol A Biol Sci Med Sci. 2022. Epub 20220414. doi: 10.1093/gerona/glac088 . 35421897 PMC9977227

[31] Gong YC, Chen X, Song QT, Gan Y, Zhang B, Li BS, et al. A randomized placebo-controlled study: Phellodendron Bawei tablets combined with standard management can improve storage symptoms, sleep quality, and medication compliance in patients with benign prostatic hyperplasia compared to placebo with standard management. Transl Androl Urol. 2021;10(8):3423–31. doi: 10.21037/tau-21-588 ; PubMed Central PMCID: PMC8421819. 34532267 PMC8421819

[32] Hou J, Zhang R. Clinical Analysis on the Effects of Tandospirone Citrate Assisted by Drawing Therapy on Medication Compliance and Sleep Quality in Patients with Anxiety Disorders. Emerg Med Int. 2022;2022:9295627. Epub 20220830. doi: 10.1155/2022/9295627 ; PubMed Central PMCID: PMC9448592. 36081955 PMC9448592

[33] Miettinen T, Sverloff J, Lappalainen OP, Linton SJ, Sipila K, Kalso E. Sleep problems in pain patients entering tertiary pain care: the role of pain-related anxiety, medication use, self-reported diseases, and sleep disorders. Pain. 2022;163(7):e812–e20. Epub 20210923. doi: 10.1097/j.pain.0000000000002497 ; PubMed Central PMCID: PMC9199106. 34561395 PMC9199106

[34] Mozer CL, Bhagat PH, Seward SA, Mason NR, Anderson SL, Byron M, et al. Optimizing Oral Medication Schedules for Inpatient Sleep: A Quality Improvement Intervention. Hosp Pediatr. 2021;11(4):327–33. Epub 20210317. doi: 10.1542/hpeds.2020-002261 ; PubMed Central PMCID: PMC8006200. 33731336 PMC8006200

[35] Yamamoto H, Nakagawa E, Kita Y, Kaga Y, Inagaki M. Effect of anti-attention-deficit hyperactivity disorder (ADHD) medication on clinical seizures and sleep EEG: A retrospective study of Japanese children with ADHD. Neuropsychopharmacol Rep. 2021;41(4):511–21. Epub 20211020. doi: 10.1002/npr2.12215 ; PubMed Central PMCID: PMC8698674. 34668641 PMC8698674

[36] Borhani F, Shafiepour Motlagh M, Ehsani AH, Rashidi Y, Maddah S, Mousavi SM. On the predictability of short-lived particulate matter around a cement plant in Kerman, Iran: machine learning analysis. Int J Environ Sci Technol (Tehran). 2022:1–14. Epub 20221109. doi: 10.1007/s13762-022-04645-3 ; PubMed Central PMCID: PMC9643923. 36405244 PMC9643923

[37] Peng J, Yang K, Tian H, Lin Y, Hou M, Gao Y, et al. The mechanisms of Qizhu Tangshen formula in the treatment of diabetic kidney disease: Network pharmacology, machine learning, molecular docking and experimental assessment. Phytomedicine. 2022;108:154525. Epub 20221106. doi: 10.1016/j.phymed.2022.154525 . 36413925

[38] Ahuja R. Comparative study of various machine learning algorithms for prediction of insomnia. Research Anthology on Machine Learning Techniques, Methods, and Applications. 2022;IGI Global:776–99.

[39] Zulfiker M, Kabir N., Biswas A. Chakraborty A., P. Predicting Insomnia Using Multilayer Stacked Ensemble Model. In International Conference on Advances in Computing and Data Sciences 2021.

[40] Siddiqui MM, Srivastava G, Saeed SH. Diagnosis of insomnia sleep disorder using short time frequency analysis of PSD approach applied on EEG signal using channel ROC-LOC. Sleep Sci. 2016;9(3):186–91. Epub 20160721. doi: 10.1016/j.slsci.2016.07.002 ; PubMed Central PMCID: PMC5241612. 28123658 PMC5241612

[41] Chen RF, Cai Y, Zhu ZH, Hou WL, Chen P, Wang J, et al. Sleep disorder as a clinical risk factor of major depression: associated with cognitive impairment. Asian J Psychiatr. 2022;76:103228. Epub 20220809. doi: 10.1016/j.ajp.2022.103228 . 35973338

[42] Chen X, Jiang F, Yang Q, Zhang P, Zhu H, Liu C, et al. Bilateral repetitive transcranial magnetic stimulation ameliorated sleep disorder and hypothalamic-pituitary-adrenal axis dysfunction in subjects with major depression. Front Psychiatry. 2022;13:951595. Epub 20220825. doi: 10.3389/fpsyt.2022.951595 ; PubMed Central PMCID: PMC9452697. 36090377 PMC9452697

[43] Gardner W, Fuchs F, Durieux L, Bourgin P, Coenen VA, Dobrossy M, et al. Slow Wave Sleep Deficits in the Flinders Sensitive Line Rodent Model of Depression: Effects of Medial Forebrain Bundle Deep-Brain Stimulation. Neuroscience. 2022;498:31–49. Epub 20220622. doi: 10.1016/j.neuroscience.2022.06.023 . 35750113

[44] Hoyos CM, Bartlett DJ, Phillips CL. Is Obstructive Sleep Apnea a Risk Factor for Depression in Coronary Artery Disease? Ann Am Thorac Soc. 2019;16(1):49–50. doi: 10.1513/AnnalsATS.201810-728ED . 30592447

[45] Huan Y, Mujun X, Xin L, Ping Z, Limei F, Aming L, et al. Short Sleep Duration as a Risk Factor for Depression, Anxiety and Fatigue in Patients with Leukemia. Neuropsychiatr Dis Treat. 2022;18:1573–82. Epub 20220729. doi: 10.2147/NDT.S362229 ; PubMed Central PMCID: PMC9346604. 35937713 PMC9346604

[46] Kazmi N, Wallen GR, Yang L, Alkhatib J, Schwandt ML, Feng D, et al. An exploratory study of pro-inflammatory cytokines in individuals with alcohol use disorder: MCP-1 and IL-8 associated with alcohol consumption, sleep quality, anxiety, depression, and liver biomarkers. Front Psychiatry. 2022;13:931280. Epub 20220811. doi: 10.3389/fpsyt.2022.931280 ; PubMed Central PMCID: PMC9405018. 36032219 PMC9405018

[47] Li M, Cui J, Xu B, Wei Y, Fu C, Lv X, et al. Sleep Disturbances and Depression Are Co-morbid Conditions: Insights From Animal Models, Especially Non-human Primate Model. Front Psychiatry. 2021;12:827541. Epub 20220125. doi: 10.3389/fpsyt.2021.827541 ; PubMed Central PMCID: PMC8821160. 35145441 PMC8821160

[48] Mungo A, Hein M, Lanquart JP, Loas G. [Atypical depression as a risk factor for obstructive sleep apnea syndrome in young adults]. Encephale. 2022;48(2):171–8. Epub 20210604. doi: 10.1016/j.encep.2021.02.018 . 34092378

[49] Rosenstrom T, Jokela M, Puttonen S, Hintsanen M, Pulkki-Raback L, Viikari JS, et al. Pairwise measures of causal direction in the epidemiology of sleep problems and depression. PLoS One. 2012;7(11):e50841. Epub 20121130. doi: 10.1371/journal.pone.0050841 ; PubMed Central PMCID: PMC3511346. 23226400 PMC3511346

[50] Ryu D, Jee HJ, Kim SY, Hwang SH, Pil GB, Jung YS. Luteolin-7-O-Glucuronide Improves Depression-like and Stress Coping Behaviors in Sleep Deprivation Stress Model by Activation of the BDNF Signaling. Nutrients. 2022;14(16). Epub 20220812. doi: 10.3390/nu14163314 ; PubMed Central PMCID: PMC9412559. 36014820 PMC9412559

[51] Stubbs B, Koyanagi A, Thompson T, Veronese N, Carvalho AF, Solomi M, et al. The epidemiology of back pain and its relationship with depression, psychosis, anxiety, sleep disturbances, and stress sensitivity: Data from 43 low- and middle-income countries. Gen Hosp Psychiatry. 2016;43:63–70. Epub 20160930. doi: 10.1016/j.genhosppsych.2016.09.008 . 27796261

[52] Sumi Y, Masuda F, Kadotani H, Ozeki Y. The prevalence of depression in isolated/idiopathic rapid eye movement sleep behavior disorder: A systematic review and meta-analysis. Sleep Med Rev. 2022;65:101684. Epub 20220809. doi: 10.1016/j.smrv.2022.101684 . 36150254

[53] Wang D, Zhao J, Zhai S, Ye H, Bu L, Fan F. Does sleep disturbance predicts posttraumatic stress disorder and depression among college students during COVID-19 lockdown? A longitudinal survey. Front Public Health. 2022;10:986934. Epub 20220913. doi: 10.3389/fpubh.2022.986934 ; PubMed Central PMCID: PMC9514232. 36176529 PMC9514232

[54] Alamir YA, Zullig KJ, Kristjansson AL, Wen S, Misra R, Montgomery-Downs H. A theoretical model of college students’ sleep quality and health-related quality of life. J Behav Med. 2022. Epub 20220813. doi: 10.1007/s10865-022-00348-9 . 35962152

[55] Prajsuchanai T, Tanphaichitr A, Hosiri T, Ungkanont K, Banhiran W, Vathanophas V, et al. Prevalence of high-risk for obstructive sleep apnea in attention deficit hyperactivity disorder children referred to psychiatry clinic and impact on quality of life. Front Psychiatry. 2022;13:926153. Epub 20220722. doi: 10.3389/fpsyt.2022.926153 ; PubMed Central PMCID: PMC9353399. 35935414 PMC9353399

[56] Costa e Silva JA, Chase M, Sartorius N, Roth T. Special report from a symposium held by the World Health Organization and the World Federation of Sleep Research Societies: an overview of insomnias and related disorders—recognition, epidemiology, and rational management. Sleep. 1996;19(5):412–6. doi: 10.1093/sleep/19.5.412 . 8843532

[57] Lemma S, Patel SV, Tarekegn YA, Tadesse MG, Berhane Y, Gelaye B, et al. The Epidemiology of Sleep Quality, Sleep Patterns, Consumption of Caffeinated Beverages, and Khat Use among Ethiopian College Students. Sleep Disord. 2012;2012:583510. Epub 20121125. doi: 10.1155/2012/583510 ; PubMed Central PMCID: PMC3581089. 23710363 PMC3581089

[58] Matricciani L, Fraysse F, Grobler AC, Muller J, Wake M, Olds T. Sleep: population epidemiology and concordance in Australian children aged 11–12 years and their parents. BMJ Open. 2019;9(Suppl 3):127–35. Epub 20190704. doi: 10.1136/bmjopen-2017-020895 ; PubMed Central PMCID: PMC6624061. 31273023 PMC6624061

[59] Salman LA, Shulman R, Cohen JB. Obstructive Sleep Apnea, Hypertension, and Cardiovascular Risk: Epidemiology, Pathophysiology, and Management. Curr Cardiol Rep. 2020;22(2):6. Epub 20200118. doi: 10.1007/s11886-020-1257-y . 31955254

[60] Su S, Li X, Xu Y, McCall WV, Wang X. Epidemiology of accelerometer-based sleep parameters in US school-aged children and adults: NHANES 2011–2014. Sci Rep. 2022;12(1):7680. Epub 20220510. doi: 10.1038/s41598-022-11848-8 ; PubMed Central PMCID: PMC9090869. 35538108 PMC9090869

[61] Urtnasan E, Park JU, Joo EY, Lee KJ. Deep Convolutional Recurrent Model for Automatic Scoring Sleep Stages Based on Single-Lead ECG Signal. Diagnostics (Basel). 2022;12(5). Epub 20220515. doi: 10.3390/diagnostics12051235 ; PubMed Central PMCID: PMC9140070. 35626390 PMC9140070

[62] Vidigal TA, Brasil EL, Ferreira MN, Mello-Fujita LL, Moreira GA, Drager LF, et al. Proposed management model for the use of telemonitoring of adherence to positive airway pressure equipment—position paper of the Brazilian Association of Sleep Medicine—ABMS. Sleep Sci. 2021;14(Spec 1):31–40. doi: 10.5935/1984-0063.20200086 ; PubMed Central PMCID: PMC8663734. 34917271 PMC8663734

[63] Ota M, Maki Y, Xu LY, Makino T. Prolonging effects of Valeriana fauriei root extract on pentobarbital-induced sleep in caffeine-induced insomnia model mice and the pharmacokinetics of its active ingredients under conditions of glycerol fatty acid ester as emulsifiers. J Ethnopharmacol. 2022;298:115625. Epub 20220813. doi: 10.1016/j.jep.2022.115625 . 35970315

[64] Bhatt SP, Guleria R, Vikram NK, Gupta AK. Non-alcoholic fatty liver disease is an independent risk factor for inflammation in obstructive sleep apnea syndrome in obese Asian Indians. Sleep Breath. 2019;23(1):171–8. Epub 20180722. doi: 10.1007/s11325-018-1678-7 . 30032465

[65] Piekarski D, Sullivan EV, Pfefferbaum A, Zahr NM. Poor subjective sleep predicts compromised quality of life but not cognitive impairment in abstinent individuals with Alcohol Use Disorder. Alcohol. 2022;103:37–43. Epub 20220721. doi: 10.1016/j.alcohol.2022.07.001 . 35870739 PMC9581497

[66] Yang S, Guo X, Liu W, Li Y, Liu Y. Alcohol as an independent risk factor for obstructive sleep apnea. Ir J Med Sci. 2022;191(3):1325–30. Epub 20210610. doi: 10.1007/s11845-021-02671-7 ; PubMed Central PMCID: PMC9135842. 34110582 PMC9135842

